# Relationships between concurrent language ability and mental health outcomes in a South African sample of 13-year-olds

**DOI:** 10.1371/journal.pone.0221242

**Published:** 2019-09-05

**Authors:** Michelle C. St Clair, Sarah Skeen, Marguerite Marlow, Mark Tomlinson

**Affiliations:** 1 Department of Psychology, University of Bath, Bath, United Kingdom; 2 Institute for Life Course Health Research, Department of Global Health, Stellenbosch University, Stellenbosch, South Africa; 3 School of Nursing and Midwifery, Queen’s University Belfast, Belfast, United Kingdom; University College Dublin, IRELAND

## Abstract

Children and adolescents with delayed or disordered language development are at increased risk of a number of negative outcomes, including social and emotional problems and mental health difficulties. Yet, in low- and middle- income countries, where risk factors for compromised language development are known to be prevalent, there is a lack of research on the association between child and adolescent language ability and mental health outcomes. This study evaluates data from a cross-sectional study in Khayelitsha, a semi-urban impoverished community near Cape Town, South Africa. To measure language ability, behaviour and mental health, adolescents aged 13 (n = 200) were assessed using the Riddles subtest of the Kaufman Assessment Battery for Children Version 2, the parent report Child Behaviour Checklist, and the self-report Moods and Feelings Questionnaire and the Self-Esteem Questionnaire. We conducted univariate and multivariate analyses to determine associations between language skills, self-esteem and mental health in this group of adolescents. Poor language ability was related to a range of concurrent adverse difficulties, such as attention deficits, self-esteem problems, social withdrawal, and depressive symptoms. Increased levels of language ability were related to better psychosocial profiles. In some cases, only individuals with a low level of language (bottom 10% of sample) were at increased risk of maladaptive outcomes. This study replicates the well-established relationship between language ability and poorer mental health found within high income countries in an upper middle-income country setting. Locally accessible support for children with reduced language ability is required, given the longer-term consequences of poorer mental health.

## Introduction

Language development is central to many aspects of children’s lives. Language skills are essential for successful communication of emotions, needs and thoughts, and to maintain relationships with others. Language also underpins the development of a range of psychological processes, such as emotional self-regulation [1], which in turn predicts a range of positive outcomes, including better interpersonal relationships and academic achievement [2]. Social cognition, or the processes used to navigate social situations, requires language skills to reflect on problems and is critical for success in interpersonal relationships [3].

When children or adolescents have delayed or disordered language development, there is a long-term negative impact on their well-being [3]. They are more likely to perform poorly at school and be unemployed as adults [4–6]. They are less likely to have good social skills and tend to exhibit withdrawn behaviour [7, 8]. Poor language skills are linked with problems in peer relationships [9] and difficulties with friendship development [10, 11].

Children with developmental language disorder (DLD; primary language difficulties in early childhood not accounted by other neurodevelopmental or intellectual disabilities) are at increased risk of developing psychiatric conditions [12, 13]. Referrals to child and psychiatric clinics include high rates of children with unidentified DLD [14, 15]. Thus, a strong association exists between DLD, whether identified or unidentified, and the presence of mental health problems. Childhood mental health problems associated with DLD include attention difficulties and internalising problems [9, 16]. During adolescence, depression and anxiety symptoms [17] and lower self-esteem [18] are also common in those with DLD. One possible explanation for the association between language and mental health problems is that children who have chronic difficulties with expressing themselves or understanding others are more likely to struggle with developing good socialisation skills [7] and emotional development generally [1]. Indeed, this is encapsulated into the Social Adaptation Model, whereby the associated difficulties are not caused directly by reduced language, but by a reaction to other people’s altered responses as a result of a child’s language limitations [19].

Reduced language ability, as evidenced by the research on DLD, appears to be a specific risk factor, but there is also evidence of a more general association between language ability and mental health and behavioural outcomes [20]. To the best of our knowledge, what has not been previously investigated is the potential resilient effect of high language ability, even within high income countries (HIC). This may be particularly beneficial for adolescents within low- and middle- income countries (LMIC), given the high levels of adversity. The possibility that increased language ability may provide an advantage in socio-emotional and behavioural development within a high risk context is an intriguing prospect.

There has been an increasing emphasis over the past decade on the burden of mental health problems in youth in LMIC [21, 22]. However, there has been less research on child language outcomes and their relationship with mental health. In the context of a generally poor education system with low resources for children with additional difficulties, a lack of support for these children may exacerbate subsequent mental health and behavioural problems. This highlights the importance of examining the link between language ability and mental health outcomes in adolescents in LMIC.

This paper reports on data from a randomised controlled trial of an intervention delivered through the antenatal period and the first six months of the infant’s life. The Thula Sana (bush baby) intervention was designed to enhance maternal sensitivity and promote secure infant attachment in a sample living in Khayelitsha, a semi-urban impoverished community near Cape Town, South Africa [23]. The original cohort was subsequently followed up when the children were 13 years old. This paper examines the relationship between concurrent language ability and psychological status in this group at age 13.

## Methods

### Participants

We conducted a randomised controlled trial of the Thula Sana intervention in Khayelitsha from 1999–2003 (Current Control Trials: ISRCTN25664149) and found significant benefits for the mother-infant relationship for women receiving the intervention [23]. Please see Table A in S1 File for an evaluation of the effect of the trial on our variables of interest in this paper. Over the period 2012–2014 we re-enrolled 333 (74.1%) of the children, now 13 years old, from the original sample of 449 mother-child pairs. For this paper, we analysed data from a subsample (n = 200) of these individuals. The original study received ethical approval from the University of Reading and the Health Sciences Faculty of the University of Cape Town. The follow-up study received ethical approval from the Health Research Ethics Committee of Stellenbosch University (S12/04/113). The follow-up study was not pre-registered.

### Procedure

For the 13-year assessments, re-enrolment strategies were put in place to locate the original sample. Alongside utilising existing contact details, these included going door-to-door in the original recruitment neighbourhoods and engaging local community structures. Some adolescents were no longer living with their biological mothers, in which case the current primary caregiver was recruited into the study and interviewed. Once participants were relocated and enrolled into the study, participants received transport to and from an assessment centre in Khayelitsha, a voucher for participation, and a meal before starting assessment procedures. Both adolescents and their caregivers were interviewed and adolescents participated in a series of assessments. For participants who were unable to travel, particularly those not based in the area, a data collector travelled to their homes to administer the assessment battery. Caregivers gave written informed consent for the original and follow-up study and the age 13 participants gave written informed assent, as approved by the ethics committees detailed above. The language assessment reported on here was introduced into the assessment battery after the study had started, with approximately 2/3 of the total follow-up sample receiving this language assessment.

Data were collected by trained and supervised data collectors with at least a high school diploma and considerable experience of data collection with vulnerable women and children. All 13-year interview data were entered into mobile phones and sent directly to a server which allowed for real-time monitoring of data quality. This application also allowed for branching or skipping to the next applicable item for a particular participant [24]. Direct assessments were video-recorded and interviews were audio-recorded and a subsample of these were reviewed by a supervisor for quality control purposes.

### Measures

#### Socio-demographic status

At baseline, mothers reported their level of education (primary school only, some secondary, secondary completed to either grade 9 or 10). At the 13 year follow up, caregivers reported on employment status, household monthly income and number of household members. The child’s caregiver was asked about their own HIV status and retrospectively reported if children had attended preschool.

#### Maternal mental health

At 13 years, caregiver depressive symptoms were assessed using the Patient Health Questionnaire-9 (PHQ-9), a nine-item screening tool for depression [25] which has been validated in South Africa with adequate reliability (α = .76) [26]. Each item was measured on a four-point scale and summed for a total score, ranging from 0–27. A dichotomous variable of depressive symptoms (9 or above) or no depressive symptoms (8 or lower) was used in this study.

#### Child psychological status

We used the parent-report 100 item Child Behaviour Checklist (CBCL) to measure emotional and behavioural problems [27]. The CBCL has been validated across multiple settings and used in South Africa with high reliability (α = .94) [28]. It is comprised of six subscales (Withdrawal, Somatic, Anxiety/depression, Attention, Delinquency, Aggressive) which are used to create two subscales “Internalising disorders” and “Externalising disorders”. Both the individual subscales and the total scores were evaluated in this analysis. We also used the short 13 item version of the self-report Moods and Feelings Questionnaire (MFQ; [29]) to measure depressive symptoms in the adolescents. The MFQ has been successfully used in South Africa to assess adolescent mental health with high reliability (α = .85) [30].

#### Self esteem

We used the self-report 42 item Self-Esteem Questionnaire (SEQ) to measure adolescents’ self-worth, previously validated in South Africa in translated forms with adequate reliability for the majority of subscales (α between .75 to .85, except sports/athletics at .67) [31]. This is a six sub-scale measurement that assesses different domains of experience for adolescents (Peer, School, Family, Sports/athletics, Body Image and Self) and an overall score. This allows us to evaluate self-worth relating to important domains, such as Body Image or Academic self-esteem, as well as evaluate global self-esteem in the overall score.

#### Child cognitive and language development

The Kaufman Assessment Battery for Children Version 2 (KABC-II) is a cognitive development assessment focused on processes needed to solve problems and specific, rather than global, constructs of learning ability [32]. The test has been used in LMIC and validated in an African setting [33]. We administered the full core battery to all adolescents, producing an overall Mental Processing Index score for each participant. For language development specifically, we used the non-core Riddles subtest to assess language ability in isiXhosa [32], one of South Africa’s official languages. For this task, the examiner says several characteristics of a concrete or abstract verbal concept, and the participant responds by naming the item.

#### Language ability

Language outcomes were considered as continuous outcomes using the raw scores of the Riddles subtest. These scores were also used to create a categorical variable of high (>26), intermediate (20–26) and low language ability (<20), with the high and low groups corresponding to best cut-off for the top and bottom 10% of the entire sample respectively.

### Statistical analysis

The analysis does not focus on the effect of the early intervention, although treatment group membership was controlled for in all analyses. Rather, we conducted analyses to determine associations between current language ability, self-esteem and mental health. This study focused on adolescents who had completed the Riddles subtest, which was administered to approximately 2/3 of the adolescents as this assessment was added after the study commenced. The missing data mechanism was missing completely at random (e.g., whether or not they received the language subtest was random), meaning that the existing data is an accurate estimation of language ability and the missing data requires no further evaluation. Please see Table B in S1 File for a comparison of the demographic variables and the outcomes across the sample who received the language assessment and the sample who did not received the language assessment.

Data were analysed using Stata 13 [34]. For each outcome measure, we evaluated the effect of a series of covariates relating to domains which have previously been associated with child mental health problems. The covariates were child gender, child age at time of testing, maternal education, preschool attendance, number of household members, and current caregiver’s household income, employment status, HIV status, and depression. The covariates that significantly predicted each outcome variable individually were included as a covariate in the final analysis. This varied for each individual outcome measurement (see supplementary materials for details of the specific covariates for each outcome measurement). Condition in the RCT (intervention vs control) was covaried in all instances, independent of significant differences in the outcome variables.

The distribution of outcome variables was evaluated for normality prior to analysis. Linear regression was not considered appropriate for variables with a non-normally distribution that was so highly skewed that the majority of individuals were on the tails of the distribution. For these variables, negative binomial regression was used instead. Robust standard errors were estimated within linear regression when the distribution was not normally distributed but not skewed sufficiently to warrant use of negative binomial regression. Robust estimation is robust against violations in parametric assumptions and was also used in cases where there were unequal variances between the three language groups. Due to the amount of multiple comparisons made in this paper, the significance level is reduced to .01. Effects with a significant level between .01 and .05 are treated as marginal effects. Pairwise deletion was used, such that the individual sample size for each analysis varied dependent on the levels of valid data for all predictors and covariates.

## Results

### Demographics

The sample was characterised by low maternal education, low household income, with only 51% of caregivers in employment and an average household of more than 3 people (see Table 1). Adolescents were approximately 50% female and had an average IQ below the expected level. Approximately half of this sample received the original trial condition.

**Table 1:** Demographic and outcome variables descriptive statistics.

Demographic Variables	% or Mean (SD)
Gender (% female)	50.5%
RCT (% intervention)	49.5%
Age (at 13)	13.23 (.60)
General IQ	78.15 (9.18)
Maternal Education	
Primary School	16.3%
Some Secondary	43.4%
9–10 Secondary	40.5%
Preschool attendance	81.1%
Household Income (at 13)	
Under 1000R	14.3%
1000R-5000R	68.9%
5000R and above	16.8%
Current Caregiver Employment	51.0%
Positive Current Caregiver HIV status	25.1%
Average size of current household	3.60 (2.12)
Current Caregiver Depression	29.3%
**Outcome Variables**	
CBCL	
Withdrawal	4.27 (4.04)
Somatic	2.62 (3.38)
Anxiety/Depression	4.39 (4.43)
Attention	4.32 (3.94)
Delinquency	2.74 (4.30)
Aggressive	6.45 (7.48)
*Externalising*	13.51 (14.39)
*Internalising*	11.28 (10.01)
Self-Esteem	
Peer	24.5 (2.88)
School	25.99 (3.36)
Family	26.74 (3.24)
Body	12.14 (1.92)
Sport	16.31 (1.98)
Self	23.91 (2.47)
*Total*	129.58 (11.21)
Depressive (MFQ)	4.74 (3.88)

### Language ability

When evaluating language as a continuous score, we found that lower language ability was related to more Attention problems (B = -.23, 95%CI(-.40, -.06), *p* < .01, h^2^ = .04). There were no other relationships between language ability and other CBCL subscales or the overall Externalising and Internalising scales, *p*s > .15. However, we did find a significant relationship between language ability and Body Self-Esteem (B = .13, 95%CI(.04,.22), *p* < .005, h^2^ = .04) and a marginally significant relationship with the “Self” Self-Esteem, (B = .16, 95%CI(.04,.28), *p* < .05, h^2^ = .04). In all cases, better language ability was related to higher levels of self-esteem in these specific domains. No other self-esteem subscale, nor the total score, was significantly associated with language ability, all *p*s>.06. There was no overall effect of continuous language ability on depressive symptoms (*p* = .14). Please see Table B in S1 File for the full results of all analyses.

### High and low language ability–resilient and risk effects

See Table 2 for a summary of the results by language grouping. Please see Table S4 in the supplementary materials for the full results of all analyses. High language ability appears to be a partial protective factor, associated with marginally significant low CBCL Withdrawal subscale scores (F(2,151) = 3.20, *p* < .05), with the high language group having marginally significantly lower Withdrawal scores than the medium language groups (B = -1.92, 95%CI(-3.43,-.40), *p* < .05, h^2^ = .03) (see Table 2). The linear relationship in the CBCL Attention subscale was confirmed in the language grouping analysis, as we found a significant group difference (F(2,145) = 9.10, *p* < .001), which was broken down into significant differences between all three groups. The high language group had fewer Attentional problems than the medium (B = -1.59, 95%CI(-2.77,-.40), *p* < .01, h^2^ = .02) and low language group (B = -3.75, 95%CI(-5.51,-1.98), *p* < .01, h^2^ = .06). There were also fewer Attentional problems in the medium group compared to the low language group (B = -2.16, 95%CI(-3.76,-.55), *p* < .01, h^2^ = .04). There were no other main effects of language group with any other CBCL subscale, all *p*s>.09.

**Table 2:** Means (SD) of the CBCL/Self Esteem Subscales and depressive symptoms by language grouping.

Measurement	High Language (n = 22)	Average Language (n = 150)	Low Language (n = 28)	Group Main Effect (*p* value)
**CBCL**				
**Withdrawal**	*2*.*59 (2*.*58)*	*4*.*55 (4*.*31)*	*4*.*07 (3*.*11)*	.*04*
Somatic*	2.5 (2.44)	2.70 (3.44)	2.33 (3.79)	.89
**Anxiety/Depression**	3.5 (3.36)	4.43 (4.61)	1.89 (4.61)	.37
**Attention**	**2.77 (1.54)**	**4.22 (4.08)**	**6.11 (3.93)**	**< .001**
Delinquency*	1.27 (1.88)	2.93 (4.73)	2.93 (2.85)	.10
Aggressive*	4.95 (5.46)	6.47 (7.87)	7.56 (6.59)	.32
Externalising*	9.0 (7.51)	13.62 (15.46)	16.59 (11.69)	.09
***Internalising***	8.59 (6.55)	11.68 (10.55)	11.30 (9.14)	.16
**Self-Esteem**				
**Peer**	25.32 (2.17)	24.44 (2.97)	24.18 (2.83)	.29
**School**	25.58 (3.58)	25.96 (3.23)	26.61 (3.86)	.43
**Family**	27.41 (3.58)	26.51 (3.12)	27.46 (3.53)	.21
**Body**	**13.45 (1.47)**	**11.96 (1.88)**	**12.07 (2.11)**	**.003**
**Sport**	17.21 (2.16)	16.11 (1.79)	16.61 (2.53)	.06
**Self**	*25*.*23 (2*.*83)*	*23*.*67 (2*.*39)*	*24*.*14 (2*.*27)*	.*02*
***Total***	134.0 (11.33)	128.65 (11.34)	131.07 (9.65)	.10
**Depressive (MFQ)**	*4*.*32 (3*.*97)*	*4*.*48 (3*.*82)*	*6*.*46 (3*.*78)*	.*02*

* indicates variables where skewed distributions required use of negative binomial regression.

The association between language groups and Body Self-Esteem was such that there was an overall difference in the groupings (F(2,196) = 6.19, *p* < .005), mainly driven by the high language grouping having much higher Body Self-Esteem than the medium (B = -1.50, 95%CI(-2.34,-.65), *p* < .005, h^2^ = .06) or low language group (marginal effect; B = -1.37, 95%CI(-2.42,-.32), *p* < .05, h^2^ = .03), which did not differ from each other (*p* = .74), suggesting that high language ability acted as a protective factor. There was also a marginal main effect of language group for the “Self” Self-Esteem subscale (F(2,190) = 3.89, *p* < .05). This was driven by the high language ability group having higher “Self” Self-Esteem than the medium language group (B = -1.70, 95%CI(-2.96,-.44), *p* < .01, h^2^ = .04) with no other group differences evident (*p*s>.13). All other group main effects in the other self-esteem subscales (and the total score) were not significant, *p*s>.10.

There was an overall marginal effect of language group on the occurrence of depressive symptoms (F(2,196) = 3.82, *p* < .05). This was driven by a marginally significant increase in symptoms between the high and low language group (B = 2.18, 95%CI(.02,4.34), *p* = .05, h^2^ = .02) and a significant difference between the medium and low language group (B = 2.03, 95%CI(.55,3.52), *p* < .01, h^2^ = .03). Overall, the results indicated that low language ability was a risk factor for increased depressive symptoms when compared to those of normal or high language ability.

## Discussion

To the best of our knowledge, this is the first study to show a relationship between psychological outcomes and adolescent language ability, specifically with attention difficulties, self-esteem and depressive symptoms in a South African context. Only the parent-reported attentional difficulties (not parent-report internalising problems) showed a consistent association with child language outcomes, although self-reported self-esteem and depressive symptoms were associated with language ability at age 13. The lack of an association with parent-reported internalising subscales is perhaps not surprising, however, as there is low concurrence between parent and self-report with more subjective experiences, such as depression and anxiety symptoms [35]. For more overt experiences, such as delinquency, attentional and withdrawal problems, parent report has been shown to have high concurrence with child report [35]. In this study, attentional difficulties were strongly related to poorer language ability, and there were more attentional difficulties as language ability decreased in both the medium and low language subgroups. This is in line with evidence showing elevated attentional problems in children with a history of DLD [36], as well as similar associations between language ability and attentional problems [37]. This association is unsurprising, as a child’s attention is likely to wander if their language ability is not sufficiently strong to allow them to keep up with increasing complex social environments. On the other hand, in HIC settings, the link between language problems and behavioural and emotional disruptions is well established, with 56.9% of children identified as Behavioural and/or Emotionally Disturbed in mainstream schools found to have previously unidentified DLD in a recent meta-analysis [38]. Thus it was expected that the current results would reveal more associations, particularly with the externalising scale and the delinquency and aggression subscales of the CBCL. This absence of these association must, however, be treated with caution, as our “low language group” was in no way a “language impaired” group. It may be that these stronger associations only occur in the context of more severe language difficulties and that attention problems have a more general association with language ability.

High language ability may be protective against the tendency to socially withdraw, and by extension, experience social difficulties. This is an intriguing finding, which has parallels in existing evidence showing more social withdrawal in children with DLD, in particular more reticence and solitary play, than children with typical language development [8]. It is of interest that the opposite pattern was not replicated–children with low language ability did not differ from those with medium language ability. The close association between language and socialisation is well known and theorised as a driving force behind language acquisition [39]. However, to our knowledge there is very little research into the advantages that high language ability offer, either in terms of socialisation advantages or in any other context. The current result, however, must be interpreted within the cultural context. The possibility that language ability in a high adversity context could be viewed as a resilient factor in promoting good social relationships is a research finding which needs further evaluation. Whether this finding can be extended to a low adversity, HIC context is another interesting line of future research.

Language ability was positively associated with self-esteem across different subscales, indicating that the higher the language ability of these adolescents, the higher their Peer, Body and “Self” self-esteem. It is of interest that these domains were either related to how the adolescent fits into their social world (Peer self-esteem) or related to their specific evaluation of their self (Body and “Self” self-esteem). These results are supported by previous research showing specific issues with peer relationships in children with DLD, as well as overall lower self-esteem in general [9, 18]. However, the pattern within the language grouping results did not support the link between lower self-esteem only in the low language groups, but rather supported the findings of high language conveying resilience, with the high language grouping having better self-esteem. Similar to the withdrawal findings, this suggests that high language ability may be a factor related to improved outcomes and better evaluation of the self in this cultural context.

Our finding that adolescents with low language abilities had elevated depressive symptoms parallels research on children with DLD in HIC, which finds that DLD not only increases depressive symptoms in children [17] but also a range of psychiatric diagnoses [12]. The nature of why adolescents with low language ability or DLD have increased rates of depression is currently not known, but this association has implications for wider adolescent outcomes. Adolescence is the time when half of adult psychiatric illnesses begin [40], so evaluating risk factors for elevated depression symptoms or disorders is of importance.

Overall, adolescents with low language ability were at specific risk for increased attentional problems, which likely have a negative influence on their education attainment [41], and levels of depressive symptoms. These findings are consistent with research in HIC contexts and implies that improving childhood language ability, potentially by improving the early language environment, may lead to fewer attentional problems, which could lead to better education attainment [41]. Indeed, research shows that early child language ability is integrally related to parental behaviour [42]. Parental training to better support language development may go some way towards improving longer term outcomes for children. Additionally, improvement of Speech and Language Therapy provision, as well as teacher training to help recognise and manage language difficulties in the classroom, could also help to improve longer term outcomes of children with reduced language ability. Indeed, understanding the link between adverse mental health outcomes and low child language ability may help teachers, parents and mental health professionals understand better the role of language in mental health difficulties [3]. Children with high language ability, however, were more sociable (less socially withdrawn) and had higher self-esteem. This indicates that, at least within a high adversity LMIC context, good language skills on entering adolescence appear to have a positive effect on a child’s social skills as well as how they view themselves. This further strengthens the case for implementing strategies to improve child language outcomes early in development.

We have discussed our results thus far in terms of the potential predictive effect of language on attentional, social, self-esteem and depressive symptoms. This is consistent with the previous literature reviewed above that show longitudinal associations, which provide stronger evidence of causal interpretations. However, the current study is cross sectional, therefore, we cannot discount the opposite pattern of findings, such that these specific outcomes could impact the ability of the adolescents to complete a verbal task. For example, attentional difficulties may interfere with their ability to concentrate on the verbal task. Additionally, adolescents with increased depressive symptoms may find it difficult to answer questions due to nerves or anxiety or lacking motivation. The cross-sectional nature of this study is such that we cannot discount these alternative explanations for our findings.

### Limitations

One limitation of the study is that the language assessment was carried out on only 200 of the 333 individuals tested at age 13. However, as we have previously indicated, the missing 133 individuals were missing completely at random, meaning that the existing data is unbiased. Another limitation is the fact that there was no standardised or adapted version of a language battery used. Instead a verbal ability subtest of the KABC-II was used to estimate adolescent language ability. This forms part of a wider problem of validated measures of language ability for LMICs. Indeed, within South Africa there are no validated measures of Xhosa language ability for adolescents available. The current assessment was a translated and adapted version of an English assessment of verbal ability.

Another limitation is the concurrent nature of the associations investigated. Ideally, we would have looked at how early language ability predicted later adolescent mental health outcomes. However, we are constrained by measures originally collected in this study, which did not include a measure of early language ability. This is hardly surprising, as this was not the focus of the initial intervention. While it is difficult to infer causality from concurrent relationships, we do feel the current study does contribute to the literature due to the lack of research in LMIC looking at the relationships between language ability and mental health outcomes. We feel these concurrent associations give motivation for future research in LMIC to investigate the longitudinal associations between language ability and mental health outcomes.

## Conclusion

To the best of our knowledge this was the first study to investigate the relationship between adolescent language ability and mental health outcomes in a low SES, high adversity South African context. Language ability in South African adolescents was related to attention difficulties, social withdrawal, self-esteem and depressive symptoms when language ability was low. These findings parallel findings within HIC contexts indicating that language ability is of importance to adolescent mental health outcomes in a LMIC context. As language development is a key early skill, there is a high likelihood that failing to meet developmental language targets is a common occurrence within this cultural context. This research indicates that intervening to improve language skills in children, potentially through parental intervention, increased Speech Language Therapy provision or teacher training, could be an important prevention strategy for improving adolescent mental health. Further research should evaluate more fully the link between language and mental health outcomes in this context.

## Supporting information

S1 File Tables with supporting information. Table A. The effect of the language intervention on the language outcome and the mental health outcomes (using the full sample). Results for an interaction between the group and the language variable on the mental health outcomes also included in this table.
Table B. Results by inclusion/exclusion in the Language Data subsample.
Table C. Full results for all regression with the continuous prediction of language ability.
(DOCX)
