# Supplementary material for: Relationships between concurrent language ability and mental health outcomes in a South African sample of 13-year-olds
Source: PLoS One. 2019 Sep 5;14(9):e0221242. doi: 10.1371/journal.pone.0221242 (PMC6728123; doi:10.1371/journal.pone.0221242)
Supplement: S1 File — Table A. The effect of the language intervention on the language outcome and the mental health outcomes (using the full sample). Results for an interaction between the group and the language variable on the mental health outcomes also included in this table. Table B. Results by inclusion/exclusion in the Language Data subsample. Table C. Full results for all regression with the continuous prediction of language ability. (DOCX) [file pone.0221242.s001.docx]

**Supplementary Materials**

**The effect of treatment status on outcomes.**

Please see Table A for the effect of the treatment status on the outcomes of interest, including the Riddles Language Measure.

Table A.

| **Measurement** | **Intervention (n = 162)** | **Control (n = 171)** | **Group Main Effect (*p* value)** | **Group Interaction Effect (*p* Value** |
| --- | --- | --- | --- | --- |
| **Language (Riddles)** | 22.61 (3.09) | 22.69 (2.87) | .84 | -- |
| **CBCL** |  |  |  |  |
| **Withdrawal** | 3.97 (3.32) | 4.23 (3.98) | *.52* | .39 |
| **Somatic** | 2.38 (2.91) | 2.80 (3.31) | *.31* | .81 |
| **Anxiety/Depression** | 4.70 (4.38) | 4.64 (4.20) | *.90* | .34 |
| **Attention** | 4.16 (3.65) | 4.32 (3.70) | *.69* | .26 |
| **Delinquency** | 2.50 (3.34) | 2.76 (4.39) | .57 | .88 |
| **Aggressive** | 5.77 (6.28) | 6.98 (7.15) | *.14* | .80 |
| ***Externalising*** | 12.43 (11.77) | 14.05 (13.79) | *.25* | .69 |
| ***Internalising*** | 11.05 (8.37) | 11.67 (9.76) | *.54* | .38 |
| **Self-Esteem** |  |  |  |  |
| **Peer** | 24.71 (3.25) | 24.79 (2.97) | *.82* | .75 |
| **School** | 26.35 (3.47) | 26.32 (3.64) | *.95* | .02* |
| **Family** | 27.44 (3.46) | 27.27 (3.12) | *.63* | .09 |
| **Body** | 12.59 (2.07) | 12.32 (1.99) | *.22* | .97 |
| **Sport** | 16.16 (2.54) | 16.01 (2.48) | .59 | .14 |
| **Self** | 24.41 (2.70) | 24.44 (2.88) | *.90* | .23 |
| ***Total*** | 131.66 (11.77) | 131.16 (12.02) | *.70* | .054 |
| **Depressive (MFQ)** | 4.15 (3.31) | 4.46 (4.03) | .45 | .27 |

*This results showed a significant prediction of language (higher language equated to lower school self-esteem) in the intervention group only, B = -.24, 95%CI(-.44,-.05), *p* <.05. There was no effect in the control group, *p* = .32.

**The effect of inclusion in the Language Data Subsample.**

Please see Table B for the effect of the inclusion in the language data subsample on the outcomes of interest.

Table B.

| **Demographic Variables** | **Current Sample (n = 200)** | **Excluded Sample (n = 133)** | **Difference** |
| --- | --- | --- | --- |
| Gender (% female) | 50.5% | 49.6% | n.s. |
| RCT (% intervention) | 49.5% | 47.4% | n.s. |
| Age (at 13) | 13.23 (.60) | 13.11 (.49) | n.s. |
| General IQ | 78.15 (9.18) | 79.33 (10.85) | n.s. |
| Maternal Education |  |  | n.s. |
| Primary School | 16.3% | 16.5% |  |
| Some Secondary | 43.4% | 45.1% |  |
| 9-10 Secondary | 40.5% | 40.3% |  |
| Preschool attendance | 81.1% | 65.1% | *p = .001, V = -.18* |
| Household Income (at 13) |  |  | n.s. |
| Under 1000R | 14.3% | 13.7% |  |
| 1000R-5000R | 68.9% | 66.4% |  |
| 5000R and above | 16.8% | 19.9% |  |
| Current Caregiver Employment | 51.0% | 51.1% | n.s. |
| Positive Current Caregiver HIV status | 25.1% | 26.8% | n.s. |
| Average size of current household | 3.60 (2.12) | 3.92 (2.11) | n.s. |
| Current Caregiver Depression | 29.3% | 27.1% | n.s. |
| **Outcome Variables** |  |  |  |
| CBCL |  |  |  |
| Withdrawal | 4.27 (4.04) | 3.85 (3.02) | n.s. |
| Somatic | 2.62 (3.38) | 2.55 (2.71) | n.s. |
| Anxiety/Depression | 4.39 (4.43) | 5.09 (4.02) | n.s. |
| Attention | 4.32 (3.94) | 4.11 (3.24) | n.s. |
| Delinquency | 2.74 (4.30) | 2.47 (3.25) | n.s. |
| Aggressive | 6.45 (7.48) | 6.29 (5.53) | n.s. |
| *Externalising* | 13.51 (14.39) | 12.88 (10.17) | n.s. |
| *Internalising* | 11.28 (10.01) | 11.48 (7.58) | n.s. |
| Self-Esteem |  |  |  |
| Peer | 24.5 (2.88) | 25.13 (3.39) | n.s. |
| School | 25.99 (3.36) | 26.86 (3.78) | *p* = .028, *d* = .25 |
| Family | 26.74 (3.24) | 28.28 (3.14) | *p* < .001, *d* = .48 |
| Body | 12.14 (1.92) | 12.92 (2.10) | *p* < .001, *d* = .39 |
| Sport | 16.31 (1.98) | 15.75 (3.12) | n.s. |
| Self | 23.91 (2.47) | 25.21 (3.06) | *p* < .001, *d* = .48 |
| *Total* | 129.58 (11.21) | 134.15 (12.38) | *p* < .005, *d* = .39 |
| Depressive (MFQ) | 4.74 (3.88) | 3.66 (3.30) | *p* < .01, *d* = -.29 |

**The full results for the continuous language predictor.**

Please see Table C or the full results, including all relevant covariates, for the regressions looking at how continuous language ability relates to the mental health outcomes.

Table C. Full results for all regression with the continuous prediction of language ability.

| **Outcome Variable/Predictor Variables** | ***B Value*** | ***95% CI*** | ***P Values*** |
| --- | --- | --- | --- |
| **CBCL - Withdrawal** |  |  |  |
| Language (Riddles) | -.12 | -.28,.05 | .16 |
| Maternal mental health | 1.38 | -10,2.86 | .07 |
| Intervention | -.12 | -1.33,1.09 | .84 |
| **CBCL - Somatic** |  |  |  |
| Language (Riddles) | .004 | -.08,.09 | .93 |
| Caregiver employment | -.64 | -1.10,-.19 | .006 |
| Maternal Mental health | .45 | -.06,97 | .09 |
| Intervention | -.18 | -.64,.29 | .46 |
| **CBCL - Anxiety/ Depression** |  |  |  |
| Language (Riddles) | -.03 | -.24,.17 | .75 |
| Preschool Attendance | -.05 | -1.61,1.51 | .95 |
| Maternal mental health | 2.36 | .67,4.06 | .007 |
| Intervention | -.002 | -1.29,1.29 | .99 |
| **CBCL - Attention** |  |  |  |
| Language (Riddles) | -.23 | -.40,-.07 | .006 |
| Maternal Education* | -- | -- | .82 |
| Household Income* | -- | -- | .64 |
| Caregiver employment | -1.07 | -2.34,.21 | .10 |
| Maternal mental health | 1.91 | .30,3.53 | .02 |
| Intervention | .05 | -1.18,1.29 | .93 |
| **CBCL - Delinquency** |  |  |  |
| Language (Riddles) | -.04 | -.13,.04 | .32 |
| maternal education | -- | -- | .73 |
| preschool attendance | -.18 | -.84,-.48 | .60 |
| Maternal mental health | .76 | .23,1.30 | .005 |
| Intervention | -.19 | -.68,.30 | .45 |
| **CBCL - Aggressive** |  |  |  |
| Language (Riddles) | .002 | -.07,.08 | .96 |
| Maternal Education | -- | -- | .93 |
| Preschool Attendance | -.22 | -.78,.33 | .43 |
| Household Income | -- | -- | .45 |
| Caregiver Employment | .03 | -.40,.47 | .89 |
| Household size | .06 | -.03,.15 | .20 |
| Maternal mental health | .46 | .01,.91 | .04 |
| Intervention | .06 | -.33,.45 | .76 |
| ***CBCL – Externalising*** |  |  |  |
| Language (Riddles) | -.03 | -.01,.02 | .25 |
| Preschool Attendance | -.11 | -.45,.22 | .51 |
| *Household Income* | -- | -- | .28 |
| Caregiver Employment | -.10 | -.42,.22 | .55 |
| Maternal Mental Health | .53 | .15,.92 | .01 |
| Intervention | .01 | -.29,.31 | .94 |
| ***CBCL - Internalising*** |  |  |  |
| Language (Riddles) | -.13 | -.51,.26 | .52 |
| Caregiver Employment | -3.46 | -6.17,-.74 | .01 |
| Maternal Mental Health | 4.40 | .87,7.94 | .02 |
| Intervention | -.70 | -3.54,2.14 | .63 |
| **Self Esteem – Peer** |  |  |  |
| Language (Riddles) | .13 | -.005,.27 | .06 |
| Household Size | .18 | -.02,.37 | .07 |
| Intervention | .46 | -.35,1.27 | .26 |
| **Self Esteem - School** |  |  |  |
| Language (Riddles) | -.07 | -.23,.10 | .43 |
| Preschool Attendance | .38 | -.87,1.62 | .55 |
| Intervention | -.30 | -1.27,.66 | .54 |
| **Self Esteem - Family** |  |  |  |
| Language (Riddles) | .88 | -.15,.17 | .88 |
| Child Age | -1.00 | -1.83,-.18 | .02 |
| Preschool Attendance | .54 | -.66,1.73 | .38 |
| Intervention | .18 | -.75,1.11 | .71 |
| **Self Esteem - Body** |  |  |  |
| Language (Riddles) | .13 | .04,.22 | .004 |
| Intervention | .31 | -.22,.83 | .25 |
| **Self esteem - Sport** |  |  |  |
| Language (Riddles) | .04 | -.05,.14 | .37 |
| Child Gender | -.48 | -1.04,.08 | .09 |
| Household Size | .01 | -.12,.14 | .89 |
| Intervention | .01 | -.55,.57 | .96 |
| **Self Esteem - Self** |  |  |  |
| Language (Riddles) | .16 | .04,.28 | .01 |
| Preschool Attendance | .21 | -.61,1.03 | .61 |
| Household Size | .17 | .01,.34 | .04 |
| Intervention | .05 | -.63,.73 | .88 |
| ***Self Esteem -Total*** |  |  |  |
| Language (Riddles) | .37 | -.14,.87 | .16 |
| Preschool Attendance | .79 | -3.21,4.79 | .70 |
| Intervention | .58 | -2.59,3.75 | .72 |
| **Depressive Symptoms (MFQ)** |  |  |  |
| Language (Riddles) | -.14 | -.33,.05 | .14 |
| Intervention | .98 | -.09,2.05 | .07 |

**The full results for the categorical language predictor.**

Please see Table S3 for the full results, including all relevant covariates, for the regressions looking at how categorical language ability relates to the mental health outcomes.

Table S4. Full results for all regression with the categorical prediction of language ability.

| **Outcome Variable/Predictor Variables** | ***B Value*** | ***95% CI*** | **P Values** |
| --- | --- | --- | --- |
| **CBCL - Withdrawal** |  |  |  |
| Language (Riddles) | -- | -- | .04 |
| Maternal mental health | 1.35 | -.13,2.84 | .07 |
| Intervention | -.12 | -1.31,1.08 | .84 |
| **CBCL - Somatic** |  |  |  |
| Language (Riddles) | -- | -- | .75 |
| Caregiver employment | -.66 | -1.05,-.27 | .001 |
| Maternal Mental health | .44 | .04,.83 | .03 |
| Intervention | -.20 | -.58,.19 | .46 |
| **CBCL - Anxiety/ Depression** |  |  |  |
| Language (Riddles) | -- | -- | .51 |
| Preschool Attendance | -.23 | -1.84,1.39 | .78 |
| Maternal mental health | 2.45 | .70,4.19 | .006 |
| Intervention | .04 | -1.25,1.33 | .95 |
| **CBCL - Attention** |  |  |  |
| Language (Riddles) | -- | -- | <.001 |
| Maternal Education* | -- | -- | .76 |
| Household Income* | -- | -- | .55 |
| Caregiver employment | -.95 | -2.21,.32 | .14 |
| Maternal mental health | 1.95 | .35,3.55 | .02 |
| Intervention | .11 | -1.10,1.32 | .85 |
| **CBCL - Delinquency** |  |  |  |
| Language (Riddles) | -- | -- | .12 |
| maternal education | -- | -- | .76 |
| preschool attendance | -.15 | -.72,-.43 | .62 |
| Maternal mental health | .71 | .23,1.20 | .004 |
| Intervention | -.17 | -.61,.27 | .45 |
| **CBCL - Aggressive** |  |  |  |
| Language (Riddles) | -- | -- | .58 |
| Maternal Education | -- | -- | .91 |
| Preschool Attendance | -.29 | -.72,.14 | .43 |
| Household Income | -- | -- | .45 |
| Caregiver Employment | .05 | -.33,.43 | .80 |
| Household size | .06 | -.031.13 | .08 |
| Maternal mental health | .50 | .09,.90 | .02 |
| Intervention | .08 | -.25,.42 | .62 |
| ***CBCL – Externalising*** |  |  |  |
| Language (Riddles) | -- | -- | <.05 |
| Preschool Attendance | -.18 | -.51,.15 | .28 |
| *Household Income* | -- | -- | .23 |
| Caregiver Employment | -.08 | -.40,.24 | .62 |
| Maternal Mental Health | .53 | .17,.88 | .004 |
| Intervention | .03 | -.26,.32 | .85 |
| ***CBCL - Internalising*** |  |  |  |
| Language (Riddles) | -- | -- | .32 |
| Caregiver Employment | -3.47 | -6.19,-.76 | .01 |
| Maternal Mental Health | 4.43 | .77,8.08 | .02 |
| Intervention | -.68 | -3.51,2.15 | .64 |
| **Self Esteem – Peer** |  |  |  |
| Language (Riddles) | -- | -- | .30 |
| Household Size | .17 | -.02,.37 | .08 |
| Intervention | .48 | -.33,1.29 | .25 |
| **Self Esteem - School** |  |  |  |
| Language (Riddles) | -- | -- | .48 |
| Preschool Attendance | .30 | -.96,1.56 | .64 |
| Intervention | -.28 | -1.25,.68 | .57 |
| **Self Esteem - Family** |  |  |  |
| Language (Riddles) | -- | -- | .40 |
| Child Age | -.92 | -1.70,-.14 | .02 |
| Preschool Attendance | .45 | -.74,1.64 | .45 |
| Intervention | .20 | -.72,1.12 | .67 |
| **Self Esteem - Body** |  |  |  |
| Language (Riddles) | -- | -- | .002 |
| Intervention | .32 | -.20,.84 | .22 |
| **Self esteem - Sport** |  |  |  |
| Language (Riddles) | -- | -- | .06 |
| Child Gender | -.45 | -1.09,.11 | .11 |
| Household Size | .03 | -.11,.16 | .69 |
| Intervention | .03 | -.53,.59 | .92 |
| **Self Esteem - Self** |  |  |  |
| Language (Riddles) | -- | -- | .02 |
| Preschool Attendance | .05 | -.80,.91 | .90 |
| Household Size | .19 | .03,.35 | .02 |
| Intervention | .11 | -.57,.79 | .75 |
| ***Self Esteem -Total*** |  |  |  |
| Language (Riddles) | -- | -- | .11 |
| Preschool Attendance | .37 | -3.75,4.48 | .86 |
| Intervention | .76 | -2.42,3.95 | .64 |
| **Depressive Symptoms (MFQ)** |  |  |  |
| Language (Riddles) | -- | -- | .02 |
| Intervention | 1.01 | -.05,2.07 | .06 |
